# Supplementary material for: Rapid point-of-care detection of BK virus in urine by an HFman probe-based loop-mediated isothermal amplification assay and a finger-driven microfluidic chip
Source: PeerJ. 2023 Mar 8;11:e14943. doi: 10.7717/peerj.14943 (PMC10007963; doi:10.7717/peerj.14943)
Supplement: Supplemental Information 1 [file peerj-11-14943-s001.doc]

**Supplementary Information**

**Rapid point-of-care detection of BK virus in urine by an HFman probe-based loop-mediated isothermal amplification assay and a finger-driven microfluidic chip**

Yongjuan Zhao ^1^, Yi Zeng ^1^, Renfei Lu ^2^, Zhiying Wang ^3^, Xiaoling Zhang ^1^, Nannan Wu ^1^, Tongyu Zhu ^4,5*^, Yang Wang ^3*^, Chiyu Zhang ^1*^

1 Shanghai Public Health Clinical Center, Fudan University, Shanghai, China

2 Clinical Laboratory, Nantong Third Hospital Affiliated to Nantong University, Nantong, China

3 Beijing Advanced Innovation Center for Biomedical Engineering, Key Laboratory for Biomechanics and Mechanobiology, School of Biological Science and Medical Engineering, Beihang University, Beijing, China

4 Shanghai Medical College, Fudan University, Shanghai, China

5 Shanghai Key Laboratory of Organ Transplantation, Zhongshan Hospital, Fudan University, Shanghai, China

Corresponding Authors:

Chiyu Zhang

No.2901, Caolang Road, Jinshan District, Shanghai 201508, China

Yang Wang

No.37, Xueyuan Road, Haidian District, Beijing 100083, China

Email address: chiyu_zhang1999@163.com (C. Zhang), w_yang89@163.com (Y. Wang)

# First authors: Y. Zhao, and Y. Zeng contributed equally to this article.

* T. Zhu, Y. Wang, and C. Zhang contributed equally to this article..

**Table S1.** Information on 5 sets of HF-LAMP primers for the detection of BKV.

| **Primer set** | **Primer name** | **Sequence (5′-3′)** | **Length**  **(nt)** | **Genomic location (nt)** |
| --- | --- | --- | --- | --- |
| BKV-1 | F3 | CAGCACAGCAAGAATTCC | 18 | 1710-1909 |
|  | B3 | CAACAGCAAAGAAGTGGAA | 19 |  |
|  | FIP | ACAGTTACAGCCTCCCACATTA-CTCCCCAATTTGAATGAGGA | 42 |  |
|  | BIP | AACTAGCATGCTTAACCTTCATGC-TGAATAGGCTTCCCTCCA | 42 |  |
|  | LB | AGGGTCACAAAAAGTACATGA | 21 |  |
| BKV-2 | F3 | GGACCTAACCTGTGGAAAT | 19 | 1745-1945 |
|  | B3 | TCATTAGCACTCCCTGCA | 18 |  |
|  | FIP | GCATGAAGGTTAAGCATGCTA-GTGGGAGGCTGTAACTGT | 39 |  |
|  | BIP | GGGTCACAAAAAGTGCATGAGC-TCTCCACCAACAGCAAAG | 40 |  |
|  | LB | GAGGTAAACCTATTCAAGGCAG | 22 |  |
| BKV-3 | F3 | AATGAATACTGACCATAAGGC | 21 | 2010-2231 |
|  | B3 | GAAACATACAGGCTATCAGC | 20 |  |
|  | FIP | CTGTCAAAGTCCCAAAATACCTAGT-ACAAAAACAATGCTTATCCAGT | 47 |  |
|  | BIP | GGAAAATGTTCCCCCAGTACTTCAT-TTTACAAAGAGGCCCCAC | 43 |  |
|  | LF | ACTCACGACCTAAGGACTAGG | 21 |  |
|  | LB | CACAGCTACCACAGTGTTGC | 20 |  |
| BKV-4 | F3 | CTATTTGGACAAAAACAATGCTT | 23 | 2031-2053 |
|  | B3 | GCTGAAACATACAGGCTATC | 20 |  |
|  | FIP | CCTGTCAAAGTCCCAAAATACCTAG-CCAGTTGAGTGCTGGATT | 43 |  |
|  | BIP | GGAAAATGTTCCCCCAGTACTTCAT-TTTACAAAGAGGCCCCAC | 43 |  |
|  | LB | -CACAGCTACCACAGTGTTGC | 20 |  |
| BKV-5 | F3 | GTTGAGTGCTGGATTCCT | 18 | 2059-2263 |
|  | B3 | TGTTAGTAAACAGGCCACAA | 20 |  |
|  | FIP | CACATGAAGTACTGGGGGAACAT-GAAAATACTAGGTATTTTGGGACTT | 48 |  |
|  | BIP | ACACAGCTACCACAGTGTTGC-AGCTGAAACATACAGGCT | 39 |  |
|  | LB | TGTGGGGCCTCTTTGTAAAGC | 21 |  |

F3/B3: outer primers; FIP/BIP: forward and reverse internal primers; LF/LB: forward and reverse loop primers.

**Table S2. Fluorescence signals of HF-LAMP combined with a finger-driven microfluidic chip**

| **ID** | **Result** | **+Pos./-Neg.** | **DNA extraction HF-LAMP**  **Tt value** | **qPCR assay 2 Ct value** | **ID** | **Result** | **+Pos./-Neg.** | **DNA extraction HF-LAMP**  **Tt value** | **qPCR assay 2 Ct value** |
| --- | --- | --- | --- | --- | --- | --- | --- | --- | --- |
| 8 | 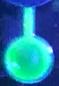 | **+** | 5.01 | 31.9 | 86 | 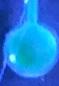 | + | 11.16 | 31.4 |
| 9 | 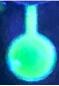 | **+** | 12.07 | 27.1 | 87 | 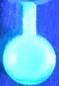 | + | 8.42 | 26.3 |
| 11 | 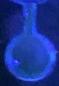 | **-** | 14.51 | 30.2 | 89 | 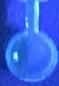 | - | 32.04 | 28.2 |
| 14 | 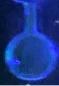 | **-** | 15.09 | 34.5 | 90 | 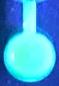 | + | 6.82 | 30.9 |
| 21 | 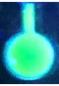 | **+** | 4.52 | 31.4 | 97 | 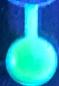 | + | 8.29 | 32.5 |
| 22 | 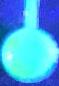 | **+** | 5.06 | 27.2 | 109 | 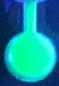 | + | 8.99 | 29.0 |
| 29 | 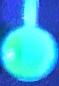 | **+** | 15.38 | 29.9 | 113 | 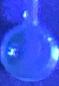 | - | 7.02 | 33.3 |
| 30 | 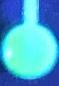 | **+** | 7.1 | 23.1 | 116 | 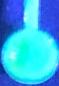 | + | 22.12 | 26.29 |
| 31 | 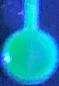 | **+** | 22.63 | 32.3 | 117 | 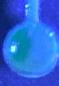 | - | Neg | Neg |
| 32 | 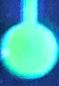 | **+** | 4.83 | 17.04 | 118 | 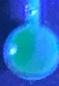 | - | Neg | Neg |
| 36 | 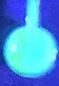 | **+** | 3.89 | 24.13 | 119 | 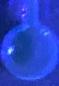 | - | Neg | Neg |
| 37 | 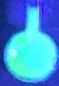 | **+** | 7.16 | 18.1 | 120 | 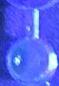 | - | Neg | Neg |
| 44 | 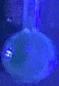 | **-** | 5.14 | 25.7 | 121 | 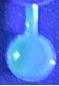 | + | Neg | Neg |
| 49 | 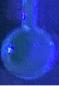 | **-** | 9.52 | 30.2 | 122 | 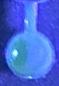 | - | Neg | Neg |
| 56 | 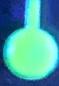 | **+** | 5.12 | 26.5 | 123 | 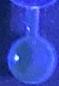 | - | Neg | Neg |
| 57 | 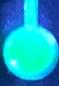 | **+** | 6.89 | 27.4 | 124 | 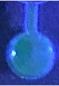 | - | Neg | Neg |
| 61 | 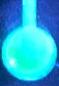 | **+** | 7.32 | 31.0 | 125 | 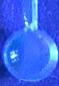 | - | Neg | Neg |
| 74 | 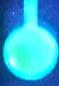 | **+** | 6.11 | 22.8 | 126 | 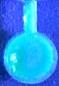 | + | Neg | Neg |
| 78 | 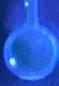 | **-** | 4.8 | 22.2 | positive control | 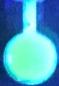 | + | Pos. | Pos. |

Pos., positive; Neg., negative. qPCR assay 2: previously reported qPCR assay

**Table S3.** Estimated cost (CN ¥) of the HF-LAMP assay.

| Component | Size | Commercial price  (¥) | HF-LAMP | Extract-free HF-LAMP |
| --- | --- | --- | --- | --- |
|  |  |  | per reaction (¥) | per reaction (¥) |
| TIANamp Micro DNA Kit | 50 tests | 768 | 15.36 | - |
| Primer and probe | - | - | 1.654 | 1.654 |
| Bst 4.0 DNA Polymerase | 16000 U | 4000 | 2.000 | 2.000 |
| NEB Q5® High-Fidelity DNA Polymerase | 500 U | 2699 | 0.807 | 0.807 |
| Vagzme dNTP Set, 100 mM Solutions | 5 mL | 350 | 0.245 | 0.245 |
| Total | - | - | 20.066 ($2.989) | 4.706($0.701) |

The isothermal amplification buffer and MgSO_4_ solution are freely provided with the Bst 4.0 DNA Polymerase.

**Supplementary figures**

**Figure S1.** Screening of the optimal primer set for BKV detection. Different colours represent different primer sets. NTC: no-template control.

**Figure S2.** Sequence alignments of the primer and probe regions of BKV. The sequences (563 sequences) were downloaded from GenBank. The small dots indicate identical bases to the topmost sequence. The numbers in parentheses show the numbers of identical sequences.

**Figure S3.** Comparison of the extraction-free HF-LAMP assay with qPCR assay 2 (using extracted DNA) in HIV-positive cohort. Heatmap shows the Tt values of the extraction-free HF-LAMP assay and the Ct values of the qPCR assay 2 for 132 clinical samples from HIV-infected patients. qPCR assays 2, the previously reported qPCR assay.

**Figure S4.** Comparison of the extraction-free HF-LAMP assay with two qPCR assays directly using urine samples in kidney cohort. Heatmap shows the Tt values or Ct values of three different BKV detection assays using 30 clinical samples from kidney transplant patients. qPCR assays 1, the commercial qPCR kit; qPCR assays 2, the previously reported qPCR assay.
